# Supplementary material for: Characterisation of Putative Outer Membrane Proteins from Leptospira borgpetersenii Serovar Hardjo-Bovis Identifies Novel Adhesins and Diversity in Adhesion across Genomospecies Orthologs
Source: Microorganisms. 2024 Jan 24;12(2):245. doi: 10.3390/microorganisms12020245 (PMC10891613; doi:10.3390/microorganisms12020245)
Supplement: Supplementary file 1 [file microorganisms-12-00245-s001.zip › Supplementary Figure S1.pdf]

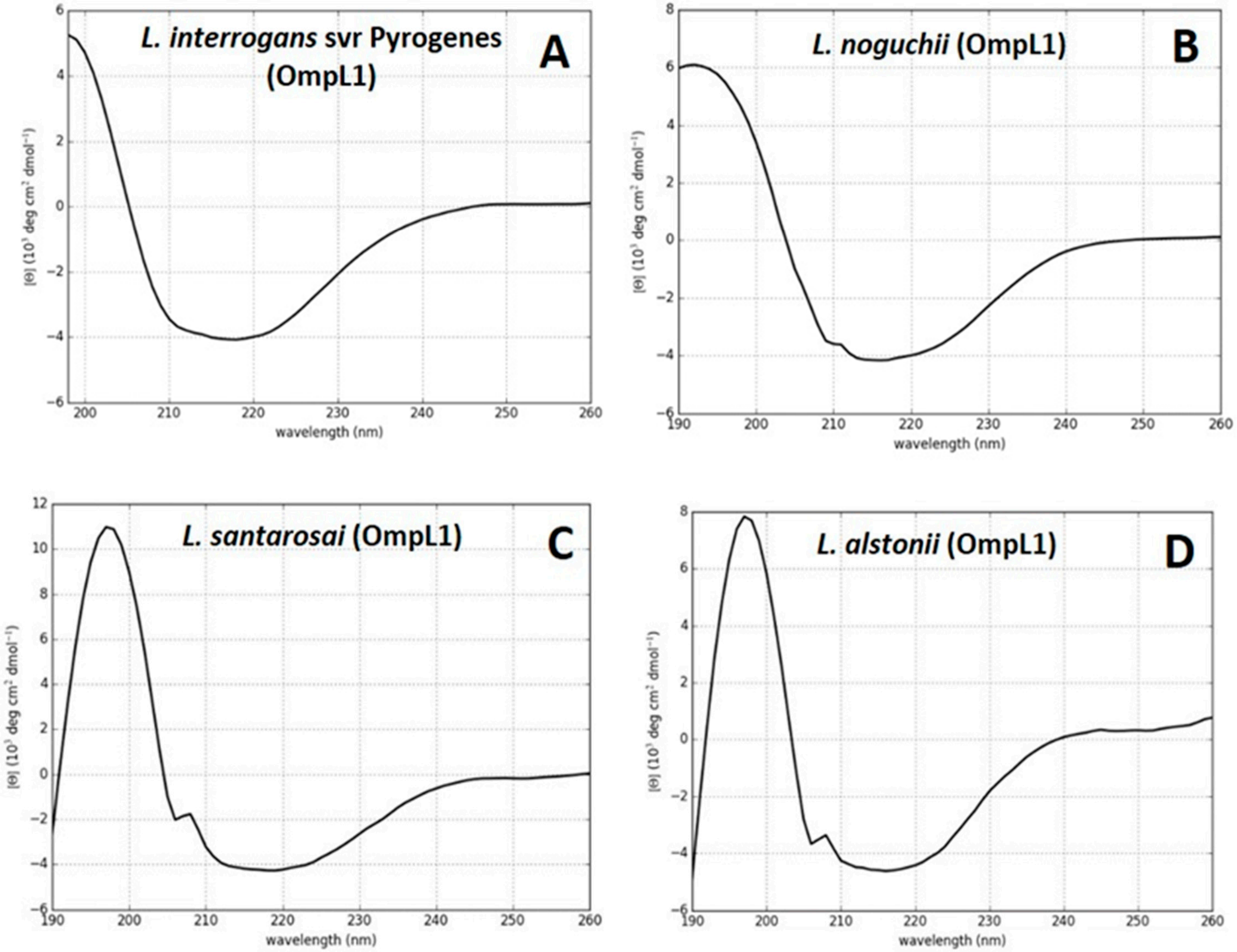

L. interrogans svr Pyrogenes (OmpL1)
A
L. noguchii (OmpL1)
B
L. santarosai (OmpL1)
C
L. alstonii (OmpL1)
D
[Θ] (10³ deg cm² dmol⁻¹)
wavelength (nm)

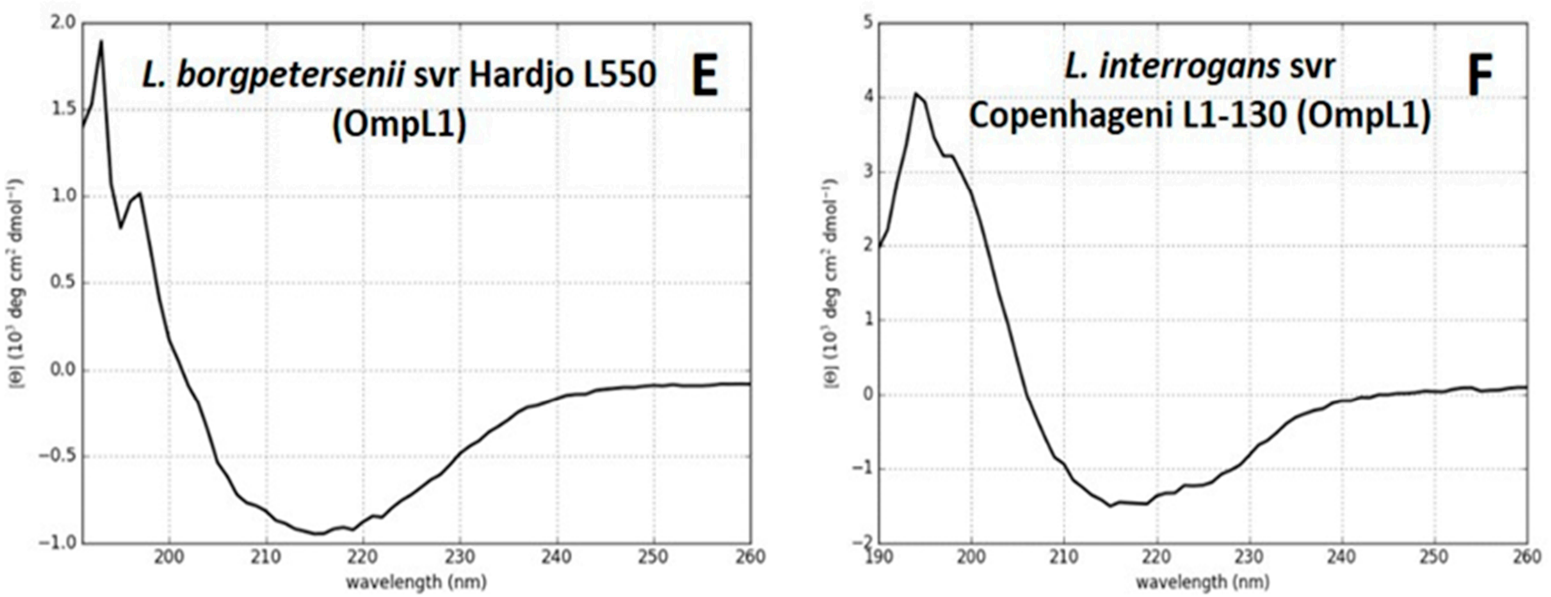

Figure S1 (A-F): Circular dichroism (CD) spectra of OmpL1 across selected genomospecies, including OmpL1 of L. borgpetersenii serovar Hardjo L550 and L. interrogans serovar Copenhageni L1-130.

All OmpL1 variants are showing a β-sheet profile with a minima band at 210 nm – 215 nm and a maxima between 195 nm – 200 nm. The CD spectrum is presented as an average of three scans recorded from 190 to 260 nm. All graphs were plotted using CAPITO software.
