# Supplementary material for: Characterisation of Putative Outer Membrane Proteins from Leptospira borgpetersenii Serovar Hardjo-Bovis Identifies Novel Adhesins and Diversity in Adhesion across Genomospecies Orthologs
Source: Microorganisms. 2024 Jan 24;12(2):245. doi: 10.3390/microorganisms12020245 (PMC10891613; doi:10.3390/microorganisms12020245)
Supplement: Supplementary file 1 [file microorganisms-12-00245-s001.zip › Supplementary Figure S2.pdf]

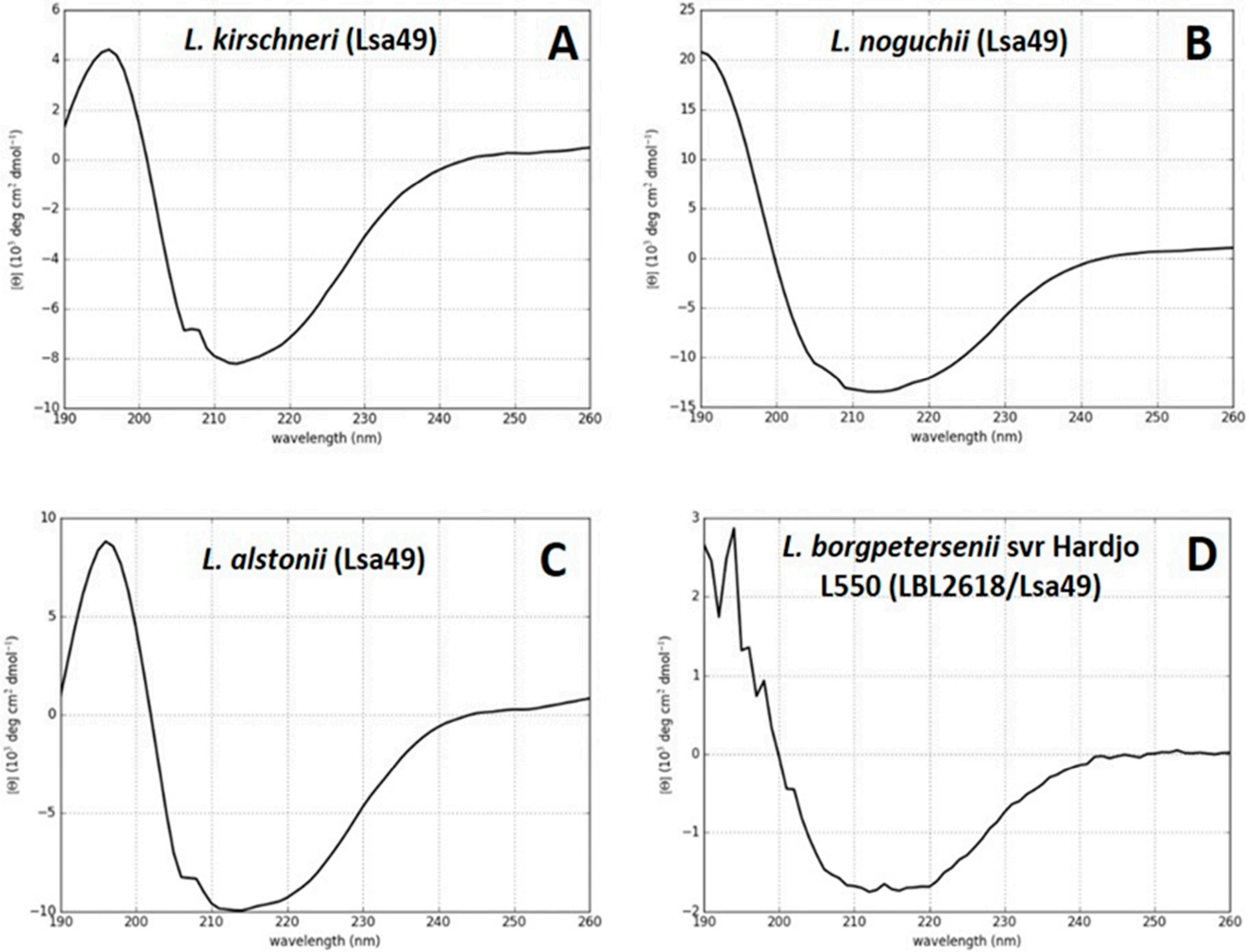

L. kirschneri (Lsa49)
A
L. noguchii (Lsa49)
B
L. alstonii (Lsa49)
C
L. borgpetersenii svr Hardjo
L550 (LBL2618/Lsa49)
D
[Θ] (10³ deg cm² dmol⁻¹)
wavelength (nm)

Figure S2 (A-D): Circular dichroism (CD) spectra of rLBL2618 across selected *Leptospira* genomospecies.

All LBL2618 variants are showing a β-sheet profile with a minimum band at 210 nm – 215 nm and a maxima band between 195 nm – 200 nm. The CD spectrum is presented as an average of three scans recorded from 190 to 260 nm. All graphs were plotted using CAPITO software.
